# Are you getting sick? Predicting influenza-like symptoms using human mobility behaviors

**DOI:** 10.1140/epjds/s13688-017-0124-6

**Published:** 2017-10-24

**Authors:** Gianni Barlacchi, Christos Perentis, Abhinav Mehrotra, Mirco Musolesi, Bruno Lepri

**Affiliations:** 1 University of Trento, Trento, Italy; 2 SKIL, Telecom Italia, Trento, Italy; 3 Fondazione Bruno Kessler, Trento, Italy; 4 University College London, London, UK; 5 The Alan Turing Institute, London, UK

**Keywords:** computational health, human mobility, predictive models

## Abstract

Understanding and modeling the mobility of individuals is of paramount importance for public health. In particular, mobility characterization is key to predict the spatial and temporal diffusion of human-transmitted infections. However, the mobility behavior of a person can also reveal relevant information about her/his health conditions. In this paper, we study the impact of people mobility behaviors for predicting the future presence of flu-like and cold symptoms (i.e. *fever*, *sore throat*, *cough*, *shortness of breath*, *headache*, *muscle pain*, *malaise*, and *cold*). To this end, we use the mobility traces from mobile phones and the daily self-reported flu-like and cold symptoms of 29 individuals from February 20, 2013 to March 21, 2013. First of all, we demonstrate that daily symptoms of an individual can be predicted by using his/her mobility trace characteristics (e.g. total displacement, radius of gyration, number of unique visited places, etc.). Then, we present and validate models that are able to successfully predict the future presence of symptoms by analyzing the mobility patterns of our individuals. The proposed methodology could have a societal impact opening the way to customized mobile phone applications, which may detect and suggest to the user specific actions in order to prevent disease spreading and minimize the risk of contagion.

## Introduction

Nowadays, we leave traces of our life events, behaviors, interests, and habits on social networks (e.g. Facebook statuses and tweets), using mobile phones and surfing the web. All this information together works as a powerful microscope that can help us to understand and predict many phenomena of our society. Hence, researchers and policy makers have the possibility to address previously unsolved problems by using these novel sources of data. A clear example comes from mobile phone records. There are almost 6 billion of mobile phone users worldwide. The world coverage has raised from 12% of the world population in 2000 up to 96% in 2014 [1], and this number even reaches 100% of population in the developed countries. These devices generate an incredible amount of data on how we daily use our mobile phone and how we interact with other people. Furthermore, they contain location data (e.g. from where a person calls) that makes people’s movements easily traceable through the antennas to which they are connected or, even better, with ad hoc applications that register the GPS tracks.

Previous studies have demonstrated the association between the health and behavioral patterns of a person, and the possibility to predict health and well-being conditions using different sources of behavioral information from social media and mobile phones. Detection of emotional states, happiness levels and depressive disorders [2–5], prediction of physical health conditions [6, 7] and stress levels [8], and modeling of influenza spreading [9–13] are some common examples of the studies carried out in this area. Interestingly, a recent work has shown that human mobility represents a good proxy for predicting people’s mental health conditions such as depressive states [5]. In this paper, we employ a similar approach to investigate the role of human mobility for predicting the physical health conditions of a person.

Knowing in advance if someone will present certain symptoms may have significant implications in terms of public health strategy and policy. For instance, specific prevention strategies can be applied: a person can be informed through an early-warning mobile application suggesting to change her/his social interactions for the next days in order to reduce further spreading of the diseases. Thus, a predictive system of health symptoms may allow public health officers to recommend specific social actions in order to minimize the risk of contagion. Moreover, this information can also represent a valuable input for epidemiological models. We can incorporate fine-grained human mobility behaviors into disease spreading models like the Global Epidemic and Mobility (GLEaM) one [14], which already makes use of socio-demographic data and of aggregated data on population mobility patterns. However, despite the importance of such applications a little effort has been put in this field, mainly because it is very difficult to have a data set which contains both self-reported health symptoms and mobility behaviors of a single individual.

In this paper, we present an initial study to investigate the effectiveness of using individual mobility behaviors for predicting the health conditions of a person. We address the challenging problem of predicting future presence of physical health symptoms such as *fever*, *sore throat*, *cough*, *shortness of breath*, *headache*, *muscle pain*, *malaise*, and *cold* by exploring the past mobility activities of an individual, thus trying to answer the following question: *can mobility behaviors be informative regarding the future health conditions of a person?*

To address this problem, we resort to the data collected during the Mobile Territorial Lab (MTL) study [15], a longitudinal living lab that has been observing the lives of more than 100 parents through multiple data sources (e.g. mobile phone data, questionnaires, experience sampling probes, etc.) for more than two years. Then, we extract a set of daily features capturing the spatio-temporal mobility patterns of a person (e.g. total distance traveled, radius of gyration of visited places, maximum displacement from home, unique number of visited places, etc.). For each individual we analyze how the mobility metrics and the presence of symptoms correlate and change over time. We also design a machine learning framework that, using past mobility behaviors, predicts the presence of flu-like and cold symptoms with a time horizon of two days ahead. To evaluate our machine learning framework, we firstly run experiments using a feature selection step (Recursive Feature Elimination (RFE) [16]). In order to select the more predictive features, we fit one of the regression models and then we rank the features (i.e. total distance) by their weight in the model. Then, once we have a comprehensive analysis of the participant’s mobility features, we use them to predict if s/he will present certain symptoms in the next days (e.g. two days ahead).

Our results show that using the mobility patterns of an individual we can obtain promising performance for our challenging prediction task. Specifically, we obtain an Area Under the Receiving Operating Characteristic Curve (AUCROC) of 0.57, a Precision score of 0.72, a Recall score of 0.84, and F1-score of 0.77 in classifying symptoms two days ahead with a Random Forest (RF) classifier.

This paper is structured as follows. Section 2 offers an overview of the related work, while Section 3 describes the data sets we used. In Section 4, we describe the methodology of our study, detailing the approach for identifying the places, the extraction of the mobility characteristics (e.g. the radius of gyration of the visited places, the unique number and the diversity of visited places, the routine index, etc.) and the learning models used for the classification tasks. Section 5 reports and discusses the results of our experiments, and finally we derive some conclusions in Section 6.

## Related work

Information on human mobility behavior derived from mobile phones has been shown to be an invaluable source to leverage within the public health domain, both at an aggregated and individual level [17]. In many cases, researchers were able to capture how massive population moves or the daily routines of individuals, and thus to study critical issues for public health like the spread of a disease or the detection of mental health problems such as depression [17].

Mobility behaviors have been captured mainly by (i) Call Detail Records (CDRs) or Mobile Network Data generated by providers, and by (ii) smartphone applications. In the case of the former, researchers are able to understand massive phenomena such as the spreading of epidemics [18, 19], mass-migration phenomena [20] or the exposure of a population to air-pollution [21]. It is worth noticing that both CDRs and Mobile Network Data are based on the cell towers of a provider, thus resulting in a coarser spatial granularity with respect to the GPS data. In addition, CDRs suffer from low temporal resolution since they are event-driven (i.e. records are created by a call/SMS trigger), while the Mobile Network Data overcome this since they are network-driven (i.e. records are generated independently of the phone usage) [17].

On the other hand, mobile applications have also started being extensively used in health and well-being domains [7, 15, 17, 22]. Many applications rely on the longitudinal monitoring of an individual outside the clinical settings, leveraging on the multiple data sources provided by the current smartphones. The major advantage of this approach is that the collection of human behavioral routines is completely unobtrusive, fine-grained (e.g. GPS signal or calls/SMSs are collected directly from the user’s device) and personalized at the individual level. In addition, the collection of potential symptoms (e.g. fever, cough, etc.) can be self-reported by using an ad-hoc mobile phone application. In this context, Fan et al. [23] proposed a hierarchical probabilistic model to simultaneously predict individuals’ physical health by understanding how flu is spread within the proximity interaction networks dynamically captured by mobile phone Bluetooth data. They tested their model both on the MIT Social Evolution [7] data set as well as on the data collected within the iEpi Study [24], where 103 students reported their symptoms and shared their Bluetooth sensor data. In the former, they succeeded in predicting one step ahead the occurrence of the symptoms, while in the latter they revealed the underlying proximity interaction network features related with flu exposure and spreading.

Previous studies have also employed mobile phone data in order to predict daily mood states [4] and stress levels [8], and to diagnose mood changes [5, 25, 26]. For instance, Canzian and Musolesi employed well-established and novel metrics to associate human mobility characteristics and depressive states [5]. Their results show that they can identify depressive states by analyzing the mobility routines of an individual and thus they can enable a continuous monitoring of her/his mental state by a therapist.

## Data

In this work we use a data set collected during the Mobile Territorial Lab (MTL) study (for a more detailed description of the study see [15]). During the MTL study, the researchers have observed the lives of more than 100 parents for almost three years (January 2013-December 2015). The participants live in the province of Trento, an area located in the Northern of Italy, and most of them are of Italian nationality. They have different levels of education (from high school diplomas to Ph.D. degrees) and types of occupation. Participants were provided with (i) an Android-based smartphone running a software able to continuously collect different mobile phone data (e.g. calls, SMSs, locations) and (ii) a survey application which is able to periodically ask the participants some questions designed by the researchers in the context of a specific study [15]. Following the Italian regulations, all participants were asked to sign informed consent forms and the study was conducted in accordance to them. The form and the MTL study were also approved by a joint Ethical Committee of University of Trento and Province of Trento.

In this paper we report a study on health symptoms that we conducted on 70 participants, 20 males and 50 females, with an age ranged from 28 to 46 (the study was run during the first phase of the MTL project when only 70 study participants were enrolled). Table 1 reports the mean and the standard deviation values of the study participants’ age.

**Table 1:** **Descriptive statistics (mean and standard deviation values) of the study participants’ age**

	**Numbers**	**Mean**	**Std.**
Men	20	39.2	3.2
Women	50	38.5	3.3

In this study, we use a combination of two type of data: (i) location data, which we use to characterize the daily mobility of the participant; and (ii) survey data with daily information about the health of the participant, which represents the ground truth of our supervised machine learning models. The data set is completely anonymized in order to ensure individuals’ privacy.

We collect symptoms data from February 20, 2013 and March 21, 2013 since in this period we have a high presence of flu-like and cold symptoms. This is also in line with the epidemic curve of the 2012-2013 influenza season, which presents a peak during our window of time [27]. In particular, we focus only on collecting one month of symptoms data in order to have a high participation rate from our study participants.

It is worth specifying that symptoms and mobility data sets do not completely overlap. This is due to the fact that there are some gaps in (i) the mobility data (i.e. participants switched off the mobile phones) and (ii) the survey data (i.e. participants did not fill the health symptoms’ survey). Hence, we have mobility data and at least one self-reported symptom for only 60 study participants.

We now describe the two different data sets that we merge by using as key the ID of the participant.

### Location data

The software installed on the smartphone continuously keeps track of: (i) the communication events (e.g. calls and SMSs), and (ii) the participant’s location captured by means of the Global Positioning System (GPS), which recorded 82% of positions with an accuracy within 20 meters [15]. In addition, to increase the number of location points we also use the position retrieved by the network provider source (i.e. the cell towers to which the phone is connected). The raw location data set consists of location point tuples $l = [\mathit{ID}, \mathit{latitude}, \mathit{longitude}, \mathit{source}, \mathit{accuracy}, \mathit{time}]$, where for each tuple *l* the study participant ID, the latitude, the longitude, the information source (i.e. GPS, Network), the accuracy of the location point in meters, and the timestamp are recorded, respectively.

Then, we employ the well-accepted notion of *mobility trace* of an individual as a set of stops and moves [5, 28]. In this notion a stop is a set of latitude and longitude points where the individual is identified to spend a particular amount of time after performing a clustering procedure,explained in Section 4.1 in detail. Formally, a stop in a place is defined as: $\mathit{Place} = [\mathit{ID}, t^{a}, t^{d}, C]$, where *ID*, $t^{a}$, $t^{d}$ and *C* stand for a place identifier, the arrival time, the departure time and the latitude-longitude coordinates, respectively. This information defines a mobility trace of places $MT(t_{1},t_{2})$ as the sequence of places visited by an individual in a given period of time: $\mathit{MT}(t_{1},t_{2}) = (Pl_{1}, Pl_{2},\ldots,Pl_{N(t_{1},t_{2})})$, where $N(t_{1},t_{2})$ is the total number of identified visited places.

### Daily health symptoms

Data on physical health symptoms were collected using a daily self-reported survey instrument, designed by an experienced epidemiologist. The survey instrument consisted of eight questions with yes/no responses for each of the following symptoms: *fever*, *sore throat*, *cough*, *shortness of breath*, *headache*, *muscle pain*, *malaise*, and *cold*.

**Figure 1:**
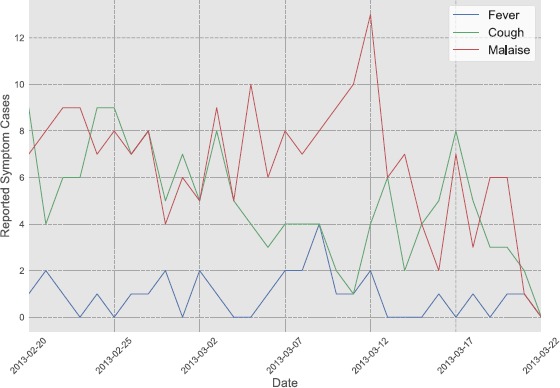
**Number of daily reported cases of fever, cough and malaise.**

Hence, the symptom raw variables have the following form: $\mathit{symptom} = [yes/no]$ In Figure 1 an example of daily reported cases for (i) fever, (ii) cough and (iii) malaise is depicted. We can notice that fever and cough have their peaks mostly in the same days. In Table 2 we report the frequencies of the eight symptoms during the entire study duration and for each symptom the number of unique individuals reporting at least one case. In the current work, we focus on all the self-reported symptoms. According with the definitions proposed by the World Health Organization (WHO) [29] and the European Centre for Disease Prevention and Control (ECDC) [30], the presence of fever, sore throat, cough, shortness of breath, headache, muscle pain, or malaise is a symptom of influenza-like illness (ILI). Cold was also chosen given the high self-reported presence of this symptom during the time period of our study.

**Table 2:** **Description of the different Symptom Types, the number of cases that were present and the unique number of individual reporting each symptom**

**Symptom Type**	**# Symptom Cases**	**# Unique Individuals**
fever	37	18
sore throat	196	40
cough	165	27
shortness of breath	86	15
headache	211	50
muscular pain	274	41
malaise	223	41
cold	174	34

The daily questions were answered at the evening by using SurveyGizmo and 64 participants, over a total of 70, reported at least one symptom.

## Methodology

Our main goal is to study the relationship between mobility behavior and self-reported symptoms. To do so, we need a set of characteristics that systematically describe human mobility behavior. Canzian et al. [5] have recently introduced metrics able to capture both presence and absence of human mobility. Such features appear to be promising in identifying physical and mental health conditions, since many of them are related with the nature of the movement. For instance, in [5] they focus on depressive symptoms which could go along with decreased movement patterns and increased spending time at home for a long-term period. In our case, we expect to identify similar signals, but in a short-term period.

### Identification of places

A very important step is the identification of places where the user is stopping. To this end, we create location clusters using raw data. In order to increase the accuracy of location estimation we consider only location points with accuracy less than 50 meters. Moreover, we ignore any location point that was collected while the user was moving. In order to infer such location points, we compute the speed of the individual by using the distance and the time between the last and the current location points. If the speed is less than a certain threshold (i.e. 5 km per hour) we consider that the location is collected when the participant was not moving.

Then, we use the location clustering approach presented in [31] in order to cluster the filtered location samples. We iterate over all location samples and for each location point we create a new cluster only if the distance of this location from the centroid of each existing cluster is more than 200 meters. Otherwise we add this location to the corresponding cluster and update its centroid.

Finally, we assign a unique place identifier to each centroid for all participants. Moreover, we assign the *home* label to the place where an individual spends the majority of the late evening and night hours (from 7 pm to 7 am), taking into account the habits in the northern part of Italy [32]. All the remaining places are labeled as *other*.

### Mobility features

For each individual, we compute all mobility features based on the visited *Places* we identify after performing the clustering procedure described above. The resulting set of mobility features is the following one:

1. *The total distance traveled* ($D_{T}(t_{1},t_{2})$): For computing the total distance traveled we consider: (i) the raw collected geo-location points when the individual is moving, and (ii) the detected stops in *Places*. We refer to them as points ${p} = [id,t^{a},t ^{d}, C]$ where $id=-1$ when the participant is moving and $id > 0$ when s/he stops in a *Place*. For a time interval $[t_{1},t_{2}]$, this mobility trace is a set $N_{p}$ of subsequent *p* points defined by a latitude-longitude pair *C*.
  1 $$ D_{T}(t_{1},t_{2}) = \sum _{i=1}^{N_{p}(t_{1},t_{2})} d(C_{i},C_{i+1}), $$
2. *The standard deviation of the total distance traveled* ($\sigma_{D_{T}}(t_{1},t_{2})$): the deviation from the average total distance (Feature 1), which is defined as:
  2 $$ \operatorname{Avg}_{D_{T}}(t_{1},t_{2}) = \frac{1}{{N_{p}(t_{1},t_{2})}-1}\sum _{i=1} ^{{N_{p}(t_{1},t_{2})}} d(C_{i},C_{i+1}). $$
  It is worth noticing that the number of movements is equal to the number of points minus 1.
3. *The total displacement* ($\operatorname{Dis}_{T}(t_{1},t_{2})$): The total displacement is a measure of the distance covered by an individual. It takes into account the distance between one *Place* where the participant stopped and the subsequent one. Formally is defined as:
  3 $$ \operatorname{Dis}_{T}(t_{1},t_{2}) = \sum _{i=1}^{N(t_{1},t_{2})} d(C_{i},C_{i+1}), $$
  where $d(C_{i},C_{i+1})$ is the geodesic distance between two visited identified places $Pl_{1}$ and $Pl_{2}$ with latitude-longitude coordinates $C_{i}$ and $C_{i+1}$, respectively.
4. *The standard deviation of the displacements* ($\sigma_{\mathrm{Dis}}(t _{1},t_{2})$): the deviation from the average displacement in $[t_{1},t_{2}]$ as defined in [5].
5. *The maximum displacement between two visited Places* ($\operatorname{Dis} _{M}(t_{1},t_{2})$): this metric quantifies the maximum displacement covered in $[t_{1},t_{2}]$.
6. *The radius of gyration of the visited Places* ($G(t_{1},t _{2})$): We measure the radius of gyration as in [5], quantifying the span of the area the participant covers. It is the deviation from the centroid of the visited places in a $[t_{1},t_{2}]$ interval weighting the contribution of each *Place* with coordinates $C_{i}$ within the set *N* by the time spent there.
  4 $$ G(t_{1},t_{2}) = \sqrt{ \frac{1}{T}\sum_{i=1}^{N(t_{1},t_{2})} T_{i} \cdot d(C_{i},C_{\mathrm{cen}})^{2}}, $$
  where $T_{i}$ equals to $t_{i}^{d}-t_{i}^{a}$ representing the time spent in the place $Pl_{i}$ and *T* is the total time spent in all the visited places in $[t_{1},t_{2}]$.
7. *The maximum displacement from Home* ($\operatorname{Dis}_{H(t_{1},t_{2})}$): this metric quantifies the maximum span the participant covered from its home. The ID and coordinates of the home for each participant is computed by considering the place with the maximum frequency of visits in *Places* considering time intervals between 7 pm-7 am, as explained in Section 4.1.
8. *The number of different Places visited* ($N_{\mathrm{dif}}(t_{1},t _{2})$): Here we simply count the number of visits in different *Places* (i.e. the number of different places where the individual had a stop) within the studied period. For example, if a participant visits within the study period $Pl_{1}$ and $Pl_{2}$ for one and two times, respectively, then the $N_{\mathrm{dif}}=3$.
9. *The number of different significant Places visited* ($N_{\mathrm{sig}}(t _{1},t_{2})$): Here, we count the number of visits in significant Places within the period under observation. We consider significant a visited place if it belongs to the *top*-10 list of the most frequent visited *Places* in the time period of the study. In Figure 2 the average number of participants’ stops over the top-N most frequent *Places* is depicted. It shows that for $N>10$ the frequencies of the stops to *Places* start to converge into a constant minimum number for our users, thus we do not consider them as significant. Therefore, we select $N=10$ as a cut-point for the significant *Places* lists.

**Figure 2:**
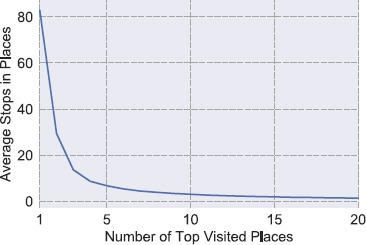
**Average number of stops in the top-N most frequent Places for the 29 participants.**
10. *The number of moving geo-location points* ($N_{\mathrm{moves}}(t_{1},t _{2})$): This is the count of the *p* geo-location points where $id={-1}$ indicating that the participant was moving in the time interval $[t_{1},t_{2}]$. It serves to quantify the moving behavior of a person.
11. *The unique number of visited Places* ($N_{\mathrm{unq}}(t_{1},t_{2})$): This feature quantifies the distinct number of stops done or places visited.
12. *The diversity of the visited Places* ($\operatorname{Div}_{\mathrm{visits}}(t_{1},t _{2})$): This metric measures how an individual spreads its visits among places in a specific time interval. This metric is a sort of entropy and was initially introduced by [33] to measure mainly the diversity in social communication, but we apply it in a spatial context. Formally it is defined as:
  5 $$ \operatorname{Div}_{\mathrm{visits}}(i) = \frac{-\sum_{j=1}^{k}v_{ij}\log {v_{ij}}}{ \log {k}}, $$
  where $v_{ij}$ is the volume of visits user *i* pays to the place *j* normalized by the total number of *i*’s visits, and *k* is the distinct number of places visited in the time interval, respectively. High values of the diversity measure indicate that participants distribute their visits more evenly among the places.
13. *Aggregated mobility features*: Previously observed mobility patterns in a participant’s historical time-line can be useful to describe the trend of the participant’s human mobility. In order to capture this information we defined a set of rolling statistics computed for each of the aforementioned mobility features. In particular, given a time window $[t_{1}, t_{2}]$ we aggregate the feature with the following statistics: mean, standard deviation, maximum, minimum and the difference of the feature values between the time $t_{1}$ and $t_{2}$.

### Classification model

We model our problem as a binary classification task, where the target variable is called *Symptom Presence* and the possible values of the label are {*Yes/No*}, that is if a user has or not at least one of the symptoms. Given a target date, our ultimate goal is to understand if a user will present or not symptoms in the forthcoming days by looking into its very recent mobility behavior. We expect to capture even slight changes in the mobility behavior (e.g. changes in covered distance) that can testify an upcoming flu and cold symptoms. Formally, given a date *t* we define:

- $t_{\mathrm{hist}}$ as the number of days we go back in individual’s historical data from the date *t*;
- historical time interval as the time interval $[t-t_{\mathrm{hist}}, t]$;
- $t_{\mathrm{hor}}$ as the number of days ahead we answer our *Symptom Presence: Yes/Not* question.

To sum up, we assign the label *Symptom Presence: Yes* to a user who presents flu-like and cold symptoms at time $t_{\mathrm{hor}}$, by using historical data in the interval $[t-t_{\mathrm{hist}}, t]$.

Due to the limited size of the data set, we decide not to built a specific model for each user. Indeed, we design a relatively general machine learning framework that can work for each user *u*. A sample for the model is built when more than three consecutive days of mobility data are available. Thus, given a date *t*, we consider valid a time window of five days if the following conditions are satisfied: (i) mobility data for $t_{\mathrm{hist}} \in [0,2]$, and (ii) symptoms data for the time $t_{\mathrm{hor}}\in [0,2]$. As mentioned in Section 3, the data set contains gaps (i.e. location points and symptoms are not available for every day). Thus, it is possible that for some samples we do not have symptoms information for all the $t_{\mathrm{hor}} \in [0,2]$. In order to keep the dimension of train/test consistent and independent from the horizon time, we created different training and test sets for each $t_{\mathrm{hor}}$. In this way, we avoid the possibility to have training samples with missing classification labels.

**Figure 3:**
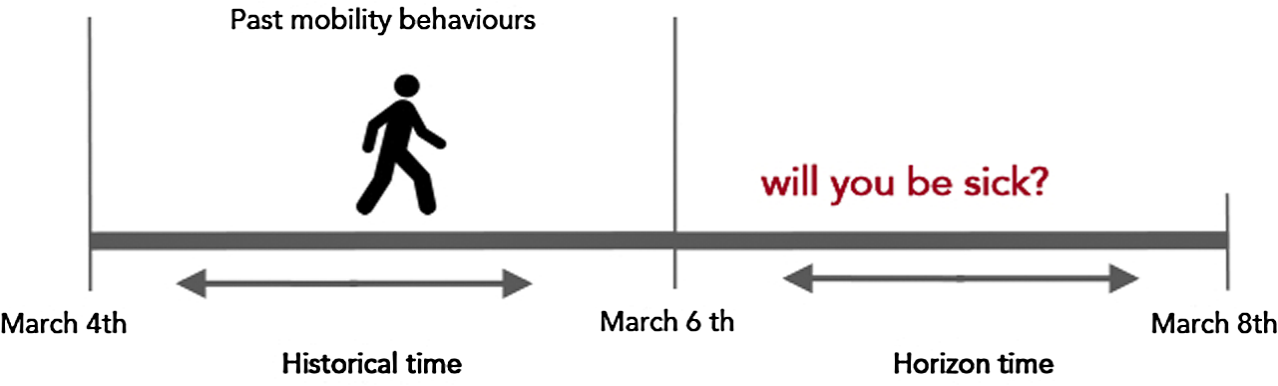
**Example of problem setting with** $\pmb{t_{\mathrm{hist}}=2}$ **and** $\pmb{t_{\mathrm{hor}}=2}$ **.**

In Figure 3 we present a toy example of the prediction task and the constraints that apply for $t_{\mathrm{hist}}=2$ and $t_{\mathrm{hor}} =2$. Given a starting day *t* (e.g. March, 6th), we impose two constraints on each participant: (i) her/his mobility data must be available from day *t* until two days back in the past (e.g. from March, 4th until March, 6th), and (ii) her/his health data must be available from day *t* up to two days in the future (e.g. from March, 6th until March, 8th). Those constraints lead to strict requirements in terms of data availability that can not always be fulfilled, because of the limitations in both data sets. Due to the aforementioned constraints we end up with a final set of 29 (12 males and 17 females) users out of 70. On the one hand the reduction of the sample size may affect the generalization of the results, but it allows us to strengthen the analysis by exposing all remaining users to equal experimental settings.

As previously said, among the symptoms described in Section 3, we classify if a user will present at least one instance of *fever*, *sore throat*, *cough*, *shortness of breath*, *headache*, *muscle pain*, *malaise*, or *cold*. Although we selected a period of the year with many cases of flu-like and cold symptoms, we dealt with a highly unbalanced data set, meaning that the dominant class is the *NO* for the *Symptom Presence* variable. We used the common approach to randomly under-sample the data set by removing samples from the over-represented class. To give an example, with $t = \mathit{Wednesday}$ we want to know if a user *u* will present flu-like and cold symptoms at $t+2 = \mathit{Friday}$ considering her/his previous mobility behaviors from the time interval $t-2 = \mathit{Monday}$, $t = \mathit{Wednesday}$.

In order to carry out our experiments, we split the data set in two parts: train and test. Then, we extract the features described in Section 4.2. For the classification task, we test four state-of-the-art machine learning models: Logistic Regression (LR) [34], Random Forest (RF) [35] and Gradient Boosted Trees (GBT) [36]. We selected these models because of their demonstrated effectiveness and, hence, popularity.

Due to the high number of features and the limited number of samples (i.e. 870 samples), we perform a feature selection step. For each classification model we evaluated several feature selection approaches by using 10-fold-cross-validation. Then, for each model we selected the best one. We found that Recursive Feature Elimination (RFE) is the best-performing feature selection method when using Logistic Regression (LR), Random Forest (RF), and Gradient Boosted Trees (GBT). We evaluate the quality of the feature selection through 10-fold-cross-validation, training the models with the reduced set of features on the training set. At this point, we can proceed with the parameters’ optimization for each model by using the selected set of features. In both, feature selection and parameters selection, we choose an optimal set in order to maximize the precision of the algorithm. The last step regards the selection of the best model. Again, through cross-validation, we train each model with its best set of features and the optimal parameters selecting the one that shows the highest precision.

## Results

In our experiments we compare three different models (LR, RF, GBT) to classify if a user will present flu-like and cold symptoms or not (i.e. fever, sore throat, cough, shortness of breath, headache, muscle pain, malaise, or cold) at a time $t_{\mathrm{hor}}$. To train our models, we use the machine learning library scikit-learn [37]. Due to the unbalanced nature of our data set, we use well-known metrics for assessing the accuracy of classification systems: (i) Precision, (ii) Recall, (iii) F1-score, and (iv) AUCROC. Precision is defined as the ratio $\frac{tp}{tp+fp}$, where *tp* is the number of true positives and *fp* is the number of false positives, while Recall is defined as the ratio $\frac{tp}{tp+fn}$, where *tp* is the number of true positives and *fn* is the number of false negatives, which are samples erroneously not labeled as belonging to the positive class. F1-score is the harmonic mean of Precision and Recall. Finally, AUCROC refers to the Area Under the Receiver Operating Characteristic curve and provides information about the ability of the models to correctly classify users with or without flu-like and cold symptoms.

### Symptoms classification

In Table 3 we present the classification results in terms of (i) Precision, (ii) Recall, (iii) F1-score, and (iv) AUCROC. We report the different performances for $t_{\mathrm{hist}} \in [-2,0] $ and $t_{\mathrm{hor}} \in [0,2]$. The results are obtained with 10-fold-cross-validation and using the best setup for each different model.

**Table 3:** **Precision (Pr.), Recall (Re.), AUCROC and F1-score of the classifiers obtained with 10-fold-cross-validation and variations of** $\pmb{t_{\mathrm{hor}}}$ **and** $\pmb{t_{\mathrm{hist}}}$

		$\boldsymbol{t_{\mathrm{hist}} = 0}$				$\boldsymbol{t_{\mathrm{hist}} = 1}$				$\boldsymbol{t_{\mathrm{hist}} = 2}$			
		**Pr.**	**AUCROC**	**Re.**	**F1**	**Pr.**	**AUCROC**	**Re.**	**F1**	**Pr.**	**AUCROC**	**Re.**	**F1**
$t_{\mathrm{hor}} = 0$	LR	0.67	0.5	0.96	0.79	0.67	0.5	1.0	0.8	0.68	0.51	1.0	0.81
	RF	0.68	0.51	0.72	0.7	0.71	0.56	0.74	0.73	0.73	0.59	0.78	0.75
	GBT	0.69	0.53	0.81	0.74	0.74	0.61	0.84	0.79	0.7	0.56	0.82	0.76
$t_{\mathrm{hor}} = 1$	LR	0.68	0.5	0.93	0.78	0.67	0.49	0.95	0.78	0.68	0.52	0.96	0.8
	RF	0.74	0.6	0.73	0.73	0.71	0.55	0.76	0.73	0.7	0.54	0.72	0.71
	GBT	0.7	0.54	0.77	0.73	0.74	0.62	0.87	0.8	0.71	0.56	0.8	0.75
$t_{\mathrm{hor}} = 2$	LR	0.68	0.51	0.99	0.81	0.68	0.51	0.91	0.78	0.68	0.5	0.95	0.79
	RF	0.71	0.55	0.76	0.73	0.73	0.58	0.72	0.73	0.72	0.57	0.74	0.73
	GBT	0.71	0.55	0.85	0.77	0.72	0.57	0.81	0.76	0.72	0.57	0.84	0.77

As expected, we observe that mobility features are relevant for predicting the presence of flu-like and cold symptoms. Interestingly, we obtain one of the best classification performance using Gradient Boosted Trees (GBT) with $t_{\mathrm{hist}}=1$ and $t_{\mathrm{hor}}=1$ (AUCROC of 0.62, a Precision score of 0.74, a Recall score of 0.87, and F1-score of 0.8). This is a consequence to the fact that people may change their mobility habits during the days before the self-reported registration of flu-like and cold symptoms, i.e. they change the mobility once they start to feel not very well. For instance, if a person is getting sick, he/she would likely go home after work instead of doing other activities.

Secondly, we can observe that as more days ahead we consider, more difficult it becomes to classify correctly the presence of symptoms by only looking at the mobility behaviors. This reveals an interesting aspect related to the fact that there is a short time period (e.g. few days) between feeling bad and reporting the symptoms. In summary, the obtained results suggest that mobility behavior can be used for our purpose, but only looking at a short period in the future (e.g. $t_{\mathrm{hor}}=2$) and considering a limited historical period. A long history of mobility data seems to be not relevant, a bigger sample size might be useful to better understand this point.

Moreover, for all the built models the following selected features (see Section 4.2) emerge as the most important ones in predicting correctly the presence of symptoms: (i) the diversity of visited places, (ii) the unique number of visited places, (iii) the number of different significant visited places, (iv) the number of moving geo-location points and (v) the aggregated mobility features. The first three features (i.e. diversity, unique number of visits and number of different significant visits) effectively capture a daily mobility routine of an individual. While the moving geo-location points quantify only the moving patterns of the participant, without considering the stops in places. Finally, the aggregated mobility features summarize the essential short-term history in people’s mobility to detect changes (i.e. the aggregated mobility behavior during the crucial days before reporting the symptoms).

To summarize, the significant features belong to three different families: (i) visited places’ routine, (ii) moving behavior and (iii) overall short-term historical mobility behavior.

For sake of completeness, we also report in Table 4 the confusion matrix for the case $t_{\mathrm{hist}}=1$ and $t_{\mathrm{hor}}=1$ using Gradient Boosted Trees (GBT), which refers to the best results in the setting of predicting future presence of flu-like and cold symptoms, i.e. one day ahead. The confusion matrix describes the performance of our classification model on the test set. We can observe that our model presents a sufficiently high accuracy in classifying the presence of symptoms while, mainly due to the difficult nature of the problem and the wide variety of symptoms we are considering, the performance with respect to the classification of the not presence of symptoms shows room for improvement.

**Table 4:** **The confusion matrix for the two-class classification task**

	**No symptoms**	**Symptoms**
**No symptoms**	0.32	0.68
**Symptoms**	0.18	0.82

Turning to the limitations of our study, we list the small number of study participants used in our analyses (i.e. 29 individuals) and the short temporal duration of our study (i.e. only 4 weeks). However, it is worth noticing that the epidemic curve of 2012-2013 influenza season presents a peak during the four weeks selected for our study. In addition, the symptoms data are self-reported by the study participants. Finally, our sample is composed by parents. Hence, it may be plausible that the predictive performance of our approach is affected also by the changes in parents’ mobility behavior related to the health status of the kids. For example, a parent may change her/his mobility behaviors in order to take the children to the doctor or to stay at home with the sick children. Moreover, the parent may get sick from her/his children, thus showing the symptoms few days later. Unfortunately, we do not collect data about the health status of the children due to privacy reasons. Therefore, future studies on different samples of study participants (e.g., students, older adults) should be conducted to better investigate the predictive role of changes in human mobility behaviors for the emergence of flu-like and cold symptoms.

## Conclusions

In this paper we have shown how to use individuals’ mobility behavior for a novel and challenging task: predicting the future presence of flu-like symptoms such as fever, cough and cold. To this end, we used the mobility information collected by mobile phones and the daily self-reported flu-like symptoms of 29 individuals in the time interval from February 20 to March 21 of 2013. Previous work has exploited the use of mobility features to predict mental health and well-being dimensions such as positive and negative emotions, stress level, and depression symptoms. To best of our knowledge, this work represents the first study that utilizes inference algorithms to predict the presence of influenza-like symptoms by only looking at the mobility behaviors of a specific individual. Our results represent a promising starting point for dealing with influenza-like public health issues. The evolution of our proposed methodology could have significant societal impact opening the way to customized mobile phone applications, which may detect the users’ condition and suggest specific actions to them in order to prevent disease spreading and minimize the risk of contagion.

In the future we plan to evaluate the predictive performance of models combining mobility information and communication interactions (e.g., number of calls, number of different contacts and so on).

## Abbreviations

MTL: Mobile Territorial Lab
AUCROC: Area Under the Receiving Operating Characteristic Curve
CDR: Call Detail Records
GPS: Global Positioning System
ECDC: European Centre for Disease Prevention and Control
ILI: Influenza-Like Illness
WHO: World Health Organization
RFE: Recursive Feature Elimination
GBT: Gradient Boosted Tree
LR: Logistic Regression
RF: Random Forest
